# Temperature controls LasR regulation of *piv* expression in *Pseudomonas aeruginosa*

**DOI:** 10.1128/mbio.00541-25

**Published:** 2025-05-20

**Authors:** Rachel E. Robinson, Joshua K. Robertson, Samantha M. Prezioso, Joanna B. Goldberg

**Affiliations:** 1 Microbiology and Molecular Genetics Program, Graduate Division of Biological and Biomedical Sciences, Laney Graduate School, Emory University 1371, Atlanta, Georgia, USA; 2 Division of Pulmonary, Asthma, Cystic Fibrosis, and Sleep, Department of Pediatrics, Emory University School of Medicine 12239, Atlanta, Georgia, USA; 3 Department of Biology, Emory University 1371, Atlanta, Georgia, USA; 4 Emory+Children's Center for Cystic Fibrosis and Airway Disease Research, Emory University School of Medicine 12239, Atlanta, Georgia, USA; University of Washington, Seattle, Washington, USA

**Keywords:** *Pseudomonas aeruginosa*, temperature, protease, quorum sensing

## Abstract

The opportunistic pathogen *Pseudomonas aeruginosa* causes debilitating lung infections in people with cystic fibrosis, as well as eye, burn, and wound infections in otherwise immunocompetent individuals. Many of *P. aeruginosa*’s virulence factors are regulated by environmental cues, such as temperature and cell density. One such virulence factor is protease IV. Prior studies have shown that *piv* expression is higher at ambient temperatures (22°C–28°C) compared to human body temperature (37°C) and also upregulated by the LasRI quorum sensing system, although it is unclear how. We found that *piv* expression was thermoregulated at stationary phase, but not exponential phase, and that *piv* is thermoregulated at the level of transcription. Using a transcriptional reporter for *piv*, we show that LasR activates *piv* expression more at 25°C at stationary phase than at 37°C. We show that key components of the LasRI quorum sensing system are not upregulated at 25°C, suggesting that LasR regulatory activity is not higher intrinsically at this temperature. We also identified sequences within the *piv* promoter that are important for its thermoregulation. We propose that LasR upregulates *piv* more at 25°C than at 37°C. The finding that temperature controls LasR regulation of *piv* highlights the complex nature of gene regulatory systems in *P. aeruginosa*.

**IMPORTANCE:**

*Pseudomonas aeruginosa* is a versatile opportunistic pathogen capable of causing many different types of infections that are often difficult to treat, such as lung infections in people with cystic fibrosis. Temperature regulates the expression of many virulence factors that contribute to *P. aeruginosa*’s ability to cause infection, yet our mechanistic understanding of virulence factor thermoregulation is poor. In this study, we show that the virulence factor protease IV is thermoregulated at the level of transcription through the quorum sensing regulator, LasR. Mechanistic studies of virulence factor thermoregulation will expand our understanding of how *P. aeruginosa* experiences different environments, including the mammalian host. Our work also highlights the importance of growth conditions in studying gene regulation, as it better elucidates the regulation of protease IV by LasR, which was previously not well understood.

## INTRODUCTION

*Pseudomonas aeruginosa* is a highly adaptable bacterial pathogen that causes lung, burn, wound, and corneal infections. To survive in the diverse niches of the human body, *P. aeruginosa* adapts to its environment and regulates the expression of many virulence factors that assist in nutrient acquisition and immune evasion. A major environmental cue that regulates virulence in many pathogenic bacteria, including *P. aeruginosa*, is the change in temperature from ambient or room temperature to that encountered in or on the human body (1, 2). Many virulence factors in *P. aeruginosa* are regulated by temperature, and mechanisms of thermoregulation have mainly been studied at the post-transcriptional (2) and post-translational levels (3–5). Thermoregulation at the level of transcription has not been as extensively studied, despite the finding that the expression of 6.4% of the genome is regulated by temperature, with many virulence factors upregulated at 37°C compared to ambient temperatures (6, 7).

In addition to temperature, another environmental cue that regulates many virulence traits in *P. aeruginosa* is the density of kin cells as sensed by the LasRI quorum sensing system (8, 9). As cell density increases, so too does the concentration of the freely diffusible homoserine lactone (HSL) autoinducer 3-oxo-C12-HSL (3O-C12-HSL) produced by the synthase LasI. At high cell density, 3O-C12-HSL complexes with the cognate transcription factor LasR to activate its function as a direct positive regulator of hundreds of genes, including many virulence and secreted factors that facilitate nutrient acquisition in resource-limited environments, such as stationary phase in *in vitro* cultures or the human body during infections (8, 10–13).

Expression of the gene encoding the virulence factor protease IV (PIV) is greatly upregulated at ambient temperatures compared to 37°C in both of the laboratory strains PAO1 and PA14 (6, 7) and is also positively regulated by LasRI quorum sensing (10–12). PIV is a secreted serine protease that degrades a broad range of human immune molecules, including the iron-sequestering lactoferrin and transferrin, complement C3, as well as alveolar surfactant proteins and IL-22 that provide protection in the lungs (14–17). It is initially translated as an ~48 kDa pre-proenzyme that contains an N-terminal signal sequence, an inactivating propeptide, and the protease domain (18). The signal sequence is cleaved during secretion, and the remaining propeptide renders the 45 kDa proenzyme catalytically inactive; release of the propeptide is required to yield the 26 kDa, enzymatically active (“mature”) protease. Cleavage of the propeptide has been reported to occur autocatalytically and by the secreted *P. aeruginosa* protease elastase (LasB) *(*15, 19, 20). PIV has been extensively characterized as a key virulence factor for *P. aeruginosa* eye infections, and strains lacking PIV are rendered avirulent in corneal infection models (21–24). Corneal infections can be difficult to treat with severe patient outcomes, as seen in a recent carbapenem-resistant *P. aeruginosa* outbreak caused by contaminated artificial tears that resulted in multiple deaths, moderate to complete loss of vision, and enucleation due to the infection *(*25).

Transcriptional regulation of *piv* is also complex, and some aspects are not fully understood. *piv* expression is directly upregulated by the sigma factor PvdS under iron starvation conditions, leading to the gene’s alternate name *prpL* (PvdS-regulated endoprotease, lysyl class) (26). It is directly repressed by the histone nucleoid structuring (H-NS) family members MvaT and MvaU, which act together to negatively regulate the same global regulon (27, 28). Although it has been well documented that *piv* expression is higher at ambient temperatures than at 37°C, a mechanism for this thermoregulation is not understood (6, 7). Additionally, while multiple transcriptomic studies have shown that *piv* is regulated by the LasRI quorum sensing system (10–12), LasR was not found to directly bind to the *piv* promoter, and thus, the mechanism by which it regulates *piv* is also not clear (29*).*

Here, we report that *piv* thermoregulation occurs at the level of transcription due to significantly higher promoter activity at room temperature (25°C) compared to human body temperature (37*°*C). Higher *piv* expression at ambient temperatures was reflected in a higher level of mature PIV protein, suggesting that temperature regulation could affect the amount of this virulence factor produced by *P. aeruginosa* experiencing different temperatures. Our work demonstrates that LasR positively regulates *piv* more at 25°C than 37*°*C and that this regulation is essential for *piv* thermoregulation. The discovery that temperature affects LasR regulation of a target gene raises the possibility that the LasRI quorum sensing may regulate certain genes disparately under conditions different from those standardly used in the laboratory.

## RESULTS

### Thermoregulation of *piv* depends on growth phase

**Fig 1:**
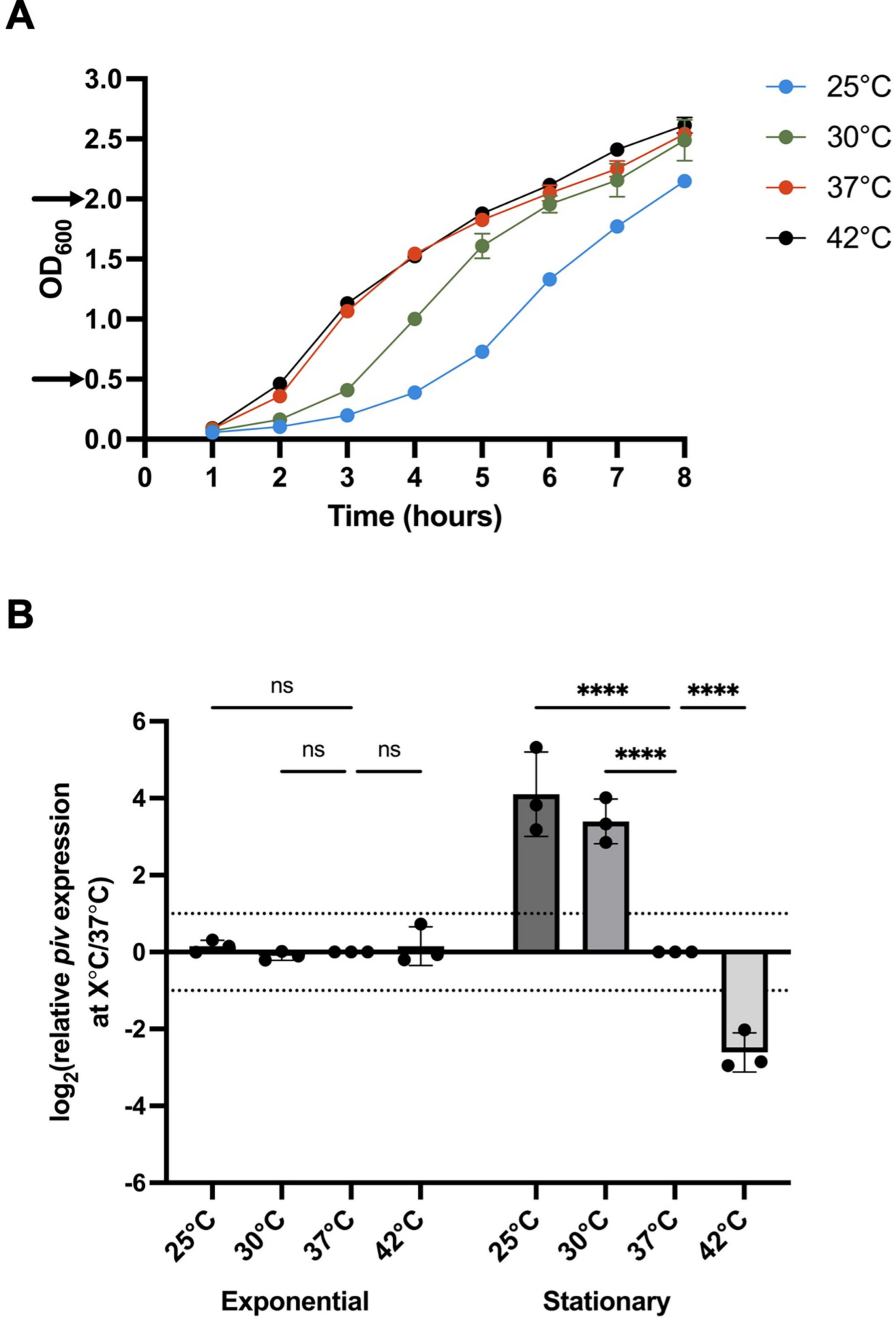
Temperature regulation of *piv* depends on growth phase. (**A**) PAO1 was grown at 25°C, 30°C, 37°C, and 42°C. RNA was extracted from an equal number of cells first at exponential phase and then from the same cultures at early stationary phase, at points during bacterial growth indicated by arrows on the *y*-axis. The average growth of three biological replicates is shown with error bars representing standard deviation. (**B**) Expression of *piv* at each temperature was calculated relative to 37°C using *omlA* as a temperature-insensitive, internal control gene. RT-qPCR was conducted in technical triplicate, and the mean of three biological replicates is shown with error bars representing standard deviation. Statistical significance was determined by two-way ANOVA with Dunnett’s multiple comparisons: *P < 0.0332, **P < 0.0021, ***P < 0.0002, and ****P < 0.0001, ns, not significant. Values between the dotted lines were not considered biologically significant.

A previous microarray study from our group showed that *piv* was more highly expressed at 22°C compared to 37°C in *P. aeruginosa* PAO1 (6). Similar results were found in an RNA-seq analysis of *P. aeruginosa* PA14 grown at 28°C compared to 37°C (7). However, both transcriptomes were determined for cells at stationary phase and only examined two temperatures: a “low” or ambient temperature and human body temperature, 37°C. We wanted to examine *piv* expression at additional temperatures to characterize if *piv* thermoregulation is binary with a “high” and “low” expression state or continuous with expression changing gradually as temperature changes. We also wanted to determine if growth phase affected *piv* thermoregulation. Wild-type *P. aeruginosa* PAO1 was grown in parallel at 25°C, 30°C, 37°C, and 42°C, and RNA was extracted from cultures at each temperature, first at exponential phase and then again from the same cultures at early stationary phase (Fig. 1A). *piv* expression at each temperature was determined relative to 37°C (a standard laboratory condition) at each growth phase using real-time quantitative PCR (RT-qPCR). Interestingly, we found that *piv* thermoregulation depended on growth phase. At exponential phase, *piv* expression was not regulated by temperature (Fig. 1B). However, at stationary phase, *piv* expression at 25°C and 30°C was ~16- and ~10-fold higher, respectively, than at 37°C and ~6-fold lower at 42°C than 37°C. These results support *piv* thermoregulation as continuous within the range of tested temperatures, with the highest expression observed at 25°C and expression decreasing gradually as temperature increases to 42°C.

**Fig 2:**
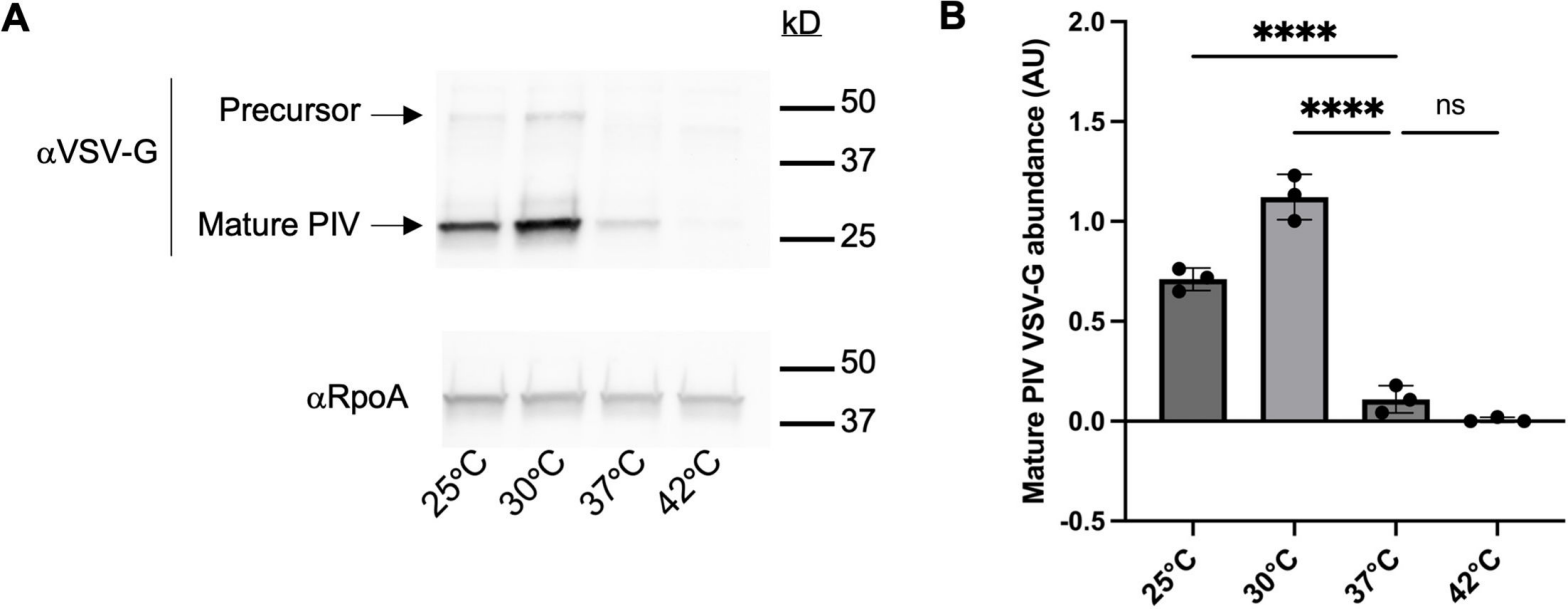
Temperature regulation of *piv* expression is reflected by levels of PIV protein. (**A**) Cell lysates of PAO1 PIV VSV-G grown to early stationary phase at 25°C, 30°C, 37°C, and 42°C were probed with antibodies against the VSV-G tag (αVSV-G) and the RNA polymerase α subunit (αRpoA). A representative image of three biological replicates is shown. (**B**) The amount of mature PIV VSV-G protein at each temperature was determined relative to the amount of RpoA using ImageJ. The mean of three biological replicates is shown with the standard deviation. Statistical significance was determined by one-way ANOVA with Dunnett’s multiple comparisons: *P < 0.0332, **P < 0.0021, ***P < 0.0002, and ****P < 0.0001, ns, not significant.

We then determined whether *piv* transcript levels were reflected in levels of PIV protein in cells grown at different temperatures. To test this, we used the strain PAO1 PIV VSV-G, in which the native *piv* locus was tagged at the C-terminus with the vesicular stomatitis virus G (VSV-G) epitope. The C-terminal placement of the VSV-G tag would allow for detecting the 48 kDa pre-proenzyme, the 45 kDa proenzyme, and the 26 kDa mature enzymatic forms of PIV, as processing of the full-length gene product involves proteolysis from the N-terminus (15, 18). PAO1 PIV VSV-G was grown at 25°C, 30°C, 37°C, and 42°C to early stationary phase, and cell lysates were subjected to quantitative immunoblotting with VSV-G antibodies (αVSV-G), as well as with RNA polymerase α subunit antibodies (αRpoA) as a temperature-independent control (Fig. 2A and B). We found that the abundance of the 26 kDa mature PIV VSV-G domain was significantly higher at 25°C and 30°C than at 37°C. Mature PIV VSV-G was hardly detected at 42°C. An unprocessed form of PIV could be detected at approximately 50 kDa at 25°C and 30°C but not at 37°C or 42°C; however, we were not able to distinguish between the 48 kDa pre-proenzyme and 45 kDa proenzyme forms of PIV. The abundance of mature PIV VSV-G at each temperature agrees well with *piv* expression data, suggesting there is likely not temperature-dependent post-transcriptional modification of *piv*. We note that densitometry quantification suggests that there is more mature PIV VSV-G protein at 30°C than at 25°C (Fig. 2B), although the difference appears more subtle when examined by visual analysis of immunoblots (Fig. 2A). This could be due to increased translation due to faster growth at 30°C compared to at 25°C (Fig. 1A). Together, these results show that *piv* thermoregulation depends on growth phase, and thermoregulation of *piv* gene expression is reflected in the abundance of PIV protein.

### *piv* expression is regulated by temperature at the level of transcription

**Fig 3:**
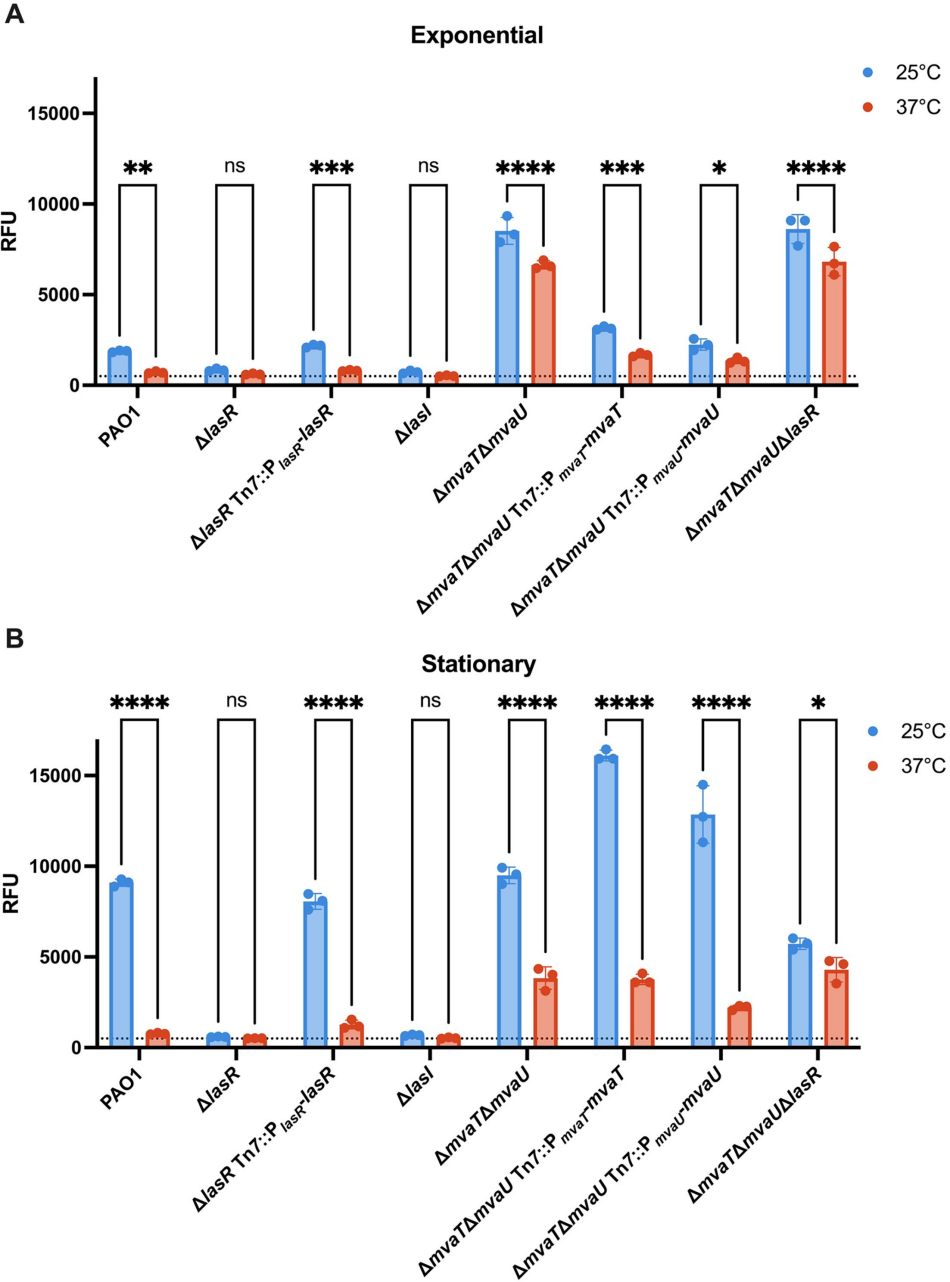
Temperature alters the activity of the *piv* promoter through the transcriptional regulator LasR, but not MvaT/MvaU. The indicated strains carrying the P*_piv_-gfp*(ASV) transcriptional reporter (pRD91) were grown at 25°C and 37°C. An equal number of cells was sampled for fluorescence (RFU) measurements (excitation 485 nm, emission 515 nm, and gain 100) first at exponential phase (**A**) and then at early stationary phase (**B**). The dotted line indicates background fluorescence of PAO1 carrying a no-promoter *gfp*(ASV) reporter plasmid (pRD87). Three biological replicates were performed, with RFU measurements conducted in technical triplicate for each. The mean of three biological replicates is shown with error bars representing standard deviation. Statistical significance was determined by two-way ANOVA with Šídák multiple comparisons: *P < 0.0332, **P < 0.0021, ***P < 0.0002, and ****P < 0.0001, ns, not significant. Statistical comparisons shown are for a given strain grown at 25°C versus 37°C.

One possible mechanism for *piv* thermoregulation could be temperature-dependent *piv* promoter activity. To test this, we made a transcriptional reporter by fusing the 200 nucleotides upstream of the *piv* coding sequence to an unstable green fluorescent protein variant, *gfp*(ASV) (30) that has been previously used for transcriptional reporters (31). PAO1 carrying the P*_piv_-gfp*(ASV) reporter plasmid (pRD91) was grown at 25°C and 37°C and assayed for fluorescence at exponential phase (Fig. 3A) and then again at stationary phase (Fig. 3B). PAO1 carrying a no-promoter *gfp*(ASV) reporter plasmid (pRD87) was used to assess background fluorescence, which was overall low at both temperatures and growth phases (Fig. S1). At both exponential and stationary phases, *piv* promoter activity in PAO1 was higher at 25°C than at 37°C, with promoter activity at 37°C not above the background (Fig. 3A and B). Promoter activity at 25°C was also higher at stationary phase than exponential phase. The finding that promoter activity was thermoregulated and affected by growth phase suggests that the growth phase-dependent thermoregulation of *piv* observed in Fig. 1 is due to differences in *piv* promoter activity.

Given that *piv* promoter activity was thermoregulated, we wondered if known transcriptional regulators of *piv* could be involved in its thermoregulation. Previous studies have shown that LasRI quorum sensing positively regulates *piv* (10–12), and the H-NS family members MvaT and MvaU both directly repress *piv* expression (27, 28). To test if LasR was required for *piv* thermoregulation, we assayed a Δ*lasR* mutant carrying the P*_piv_-gfp*(ASV) reporter for fluorescence at 25°C and 37°C as described; there was no promoter activity above background levels at either 25°C or 37°C, at both exponential and stationary phases (Fig. 3A and B). Expressing LasR in single copy from the ectopic Tn*7* site complemented the Δ*lasR* mutation, restoring wild-type *piv* promoter thermoregulation. This shows that LasR is required for the *piv* promoter activity at both 25°C and 37°C. To further confirm that *piv* was regulated by LasRI quorum sensing, we deleted the gene encoding the LasI autoinducer synthase and assayed the Δ*lasI* mutant for activity from the *piv* transcriptional reporter (Fig. 3A and B). Promoter activity in the Δ*lasI* mutant phenocopied that of the Δ*lasR* mutant, supporting *piv* being regulated by LasRI quorum sensing.

In the Δ*mvaT*Δ*mvaU* mutant, promoter activity at exponential phase was overall higher at both temperatures than in PAO1 and was slightly thermoregulated; at stationary phase, promoter activity was highly thermoregulated (Fig. 3). Although MvaT and MvaU were not required for thermoregulation, we noted that loss of MvaT and MvaU resulted in an increase in *piv* promoter activity compared to PAO1 at stationary phase at 37°C but not at 25°C. Expressing either MvaT or MvaU in single copy from the ectopic Tn*7* site complemented the Δ*mvaT*Δ*mvaU* mutation, consistent with MvaT and MvaU being functionally redundant regulators of *piv* (27, 28). Since promoter activity is thermoregulated at stationary phase in the Δ*mvaT*Δ*mvaU* mutant, this result indicates that MvaT and MvaU are not required for *piv* thermoregulation.

To test if LasR was responsible for thermoregulation observed in the Δ*mvaT*Δ*mvaU* mutant and assess the role of LasR in *piv* thermoregulation in the absence of these strong repressors, we deleted *lasR* from the Δ*mvaT*Δ*mvaU* strain. At exponential phase, promoter activity in Δ*mvaT*Δ*mvaU*Δ*lasR* was overall derepressed and slightly thermoregulated, phenocopying Δ*mvaT*Δ*mvaU* (Fig. 3A and B). However, at stationary phase, promoter activity in Δ*mvaT*Δ*mvaU*Δ*lasR* was significantly less thermoregulated due to a decrease in *piv* promoter activity at 25°C and no change at 37°C. Thus, deleting *lasR* from Δ*mvaT*Δ*mvaU* revealed that LasR only increases promoter activity at stationary phase at 25°C and that LasR does not seem to regulate *piv* at 37°C. Collectively, these results reveal that *piv* thermoregulation is occurring due to temperature-dependent promoter activity, which requires the transcriptional regulator LasR.

### LasR regulatory activity is not intrinsically higher at 25°C than at 37°C

**Fig 4:**
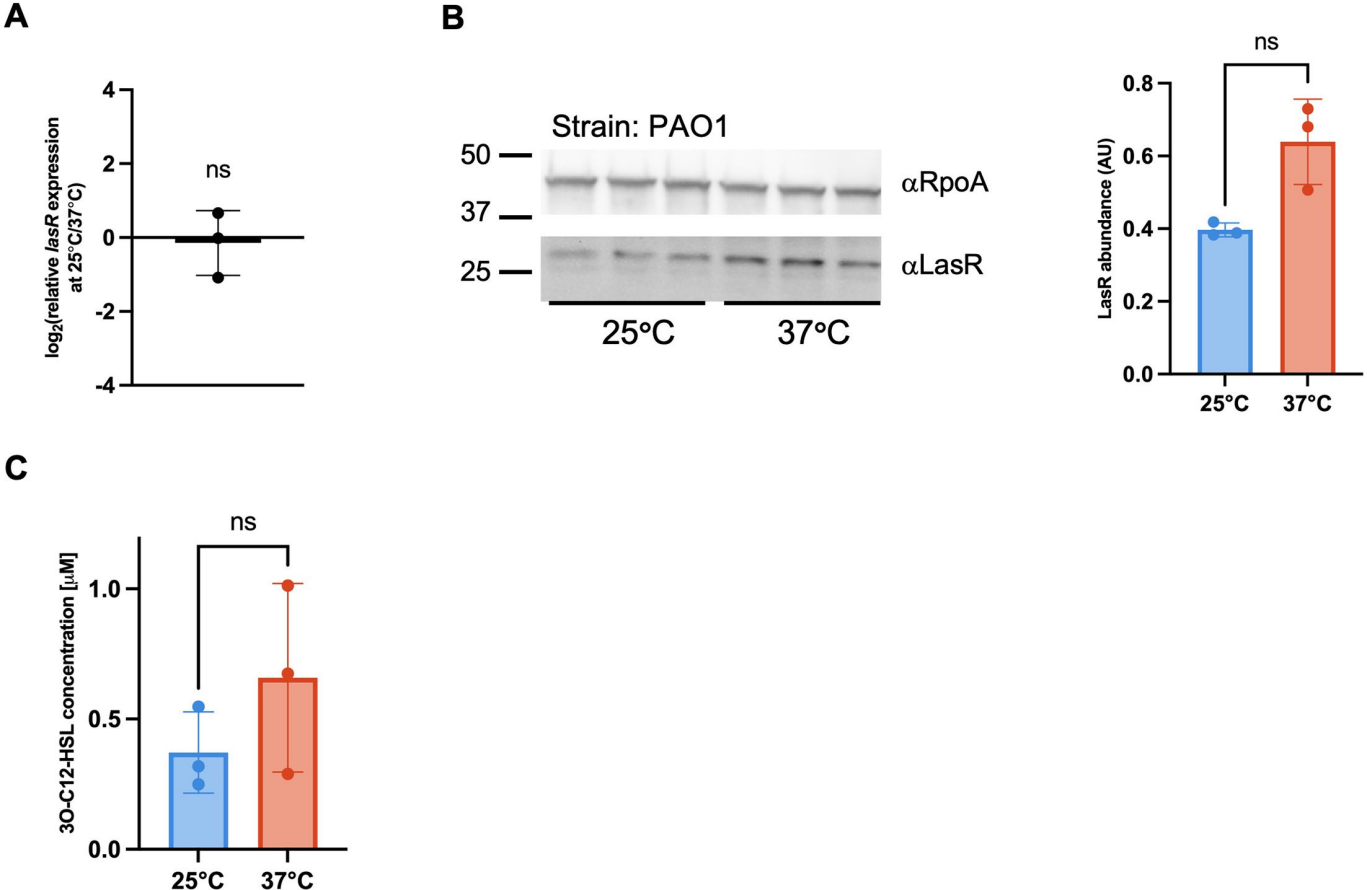
LasRI quorum sensing regulation is not higher at 25°C than at 37°C. (**A**) PAO1 was grown at 25°C and 37°C, and RNA was extracted at stationary phase as previously described. Expression of *lasR* was determined by RT-qPCR using *omlA* as an internal control gene and calculated for 25°C relative to 37°C. RT-qPCR was conducted in technical triplicate. The mean of three biological replicates analyzed is shown with error bars representing standard deviation. Statistical significance was determined by one sample *t*-test with the hypothetical value = 0 for no thermoregulation; ns, not significant. (**B**) Immunoblots (left) with αLasR antibodies and subsequent quantification (right) of LasR protein levels in PAO1 grown to stationary phase at 25°C and 37°C. All samples were probed with αRpoA as a loading control. Biological triplicates for each protein are shown. For densitometry analysis, the amount of LasR was determined relative to the amount of RpoA in the same sample using ImageJ. The mean of three biological replicates is shown with the standard deviation. Statistical significance was determined by a paired two-tailed *t* test. (**C**) PAO1 was grown at 25°C and 37°C to stationary phase, and HSLs were extracted. 3O-C12-HSL concentration was determined using an *Escherichia coli* biosensor strain that responds specifically to 3O-C12-HSL and quantified with a standard curve of commercially sourced 3O-C12-HSL.

LasR is not known to regulate its target genes in a temperature-sensitive manner, so a possible mechanism for how LasR could thermoregulate *piv* was not immediately apparent. As LasR is a positive regulator, one potential mechanism for *piv* thermoregulation by LasR could be that more LasR protein is present at 25°C than at 37°C and/or more 3O-C12-HSL is present at 25*°*C than at 37*°*C, which could lead to increased LasR activity at the lower temperature. To test this, we first examined *lasR* expression in PAO1 grown to stationary phase at 25°C and 37°C, the growth phase at which *piv* was most thermoregulated, and the phase at which LasR regulates target genes. We found that *lasR* expression was not thermoregulated (Fig. 4A), which is consistent with other studies that did not identify the *lasR* gene as being thermoregulated (6, 7, 32). We also examined the level of LasR protein in cells grown to stationary phase at 25°C and 37°C by quantitative immunoblotting with rabbit antibodies raised to LasR (12) and found that LasR protein was not more abundant at 25°C (Fig. 4B). Finally, we measured the levels of the autoinducer 3O-C12-HSL in cultures of PAO1 grown to stationary phase at 25*°*C and 37*°*C and found slightly higher levels of 3O-C12-HSL at 37*°*C than at 25*°*C (Fig. 4C). Overall, these results support that LasR protein abundance and/or regulatory activity is not higher at 25*°*C than at 37*°*C and thus is not a potential mechanism for how LasR thermoregulates *piv*.

Although MvaT and MvaU are not required for *piv* thermoregulation, we noticed that *piv* promoter activity was derepressed in Δ*mvaT*Δ*mvaU* at stationary phase at 37°C but not at 25°C. Because of this, we also checked temperature-dependent expression of *mvaT* and *mvaU* genes and protein levels of MvaT and MvaU (Fig. S2). Neither *mvaT* nor *mvaT* gene expression or protein levels were thermoregulated.

### Identification of promoter elements important for thermoregulation

**Fig 5:**
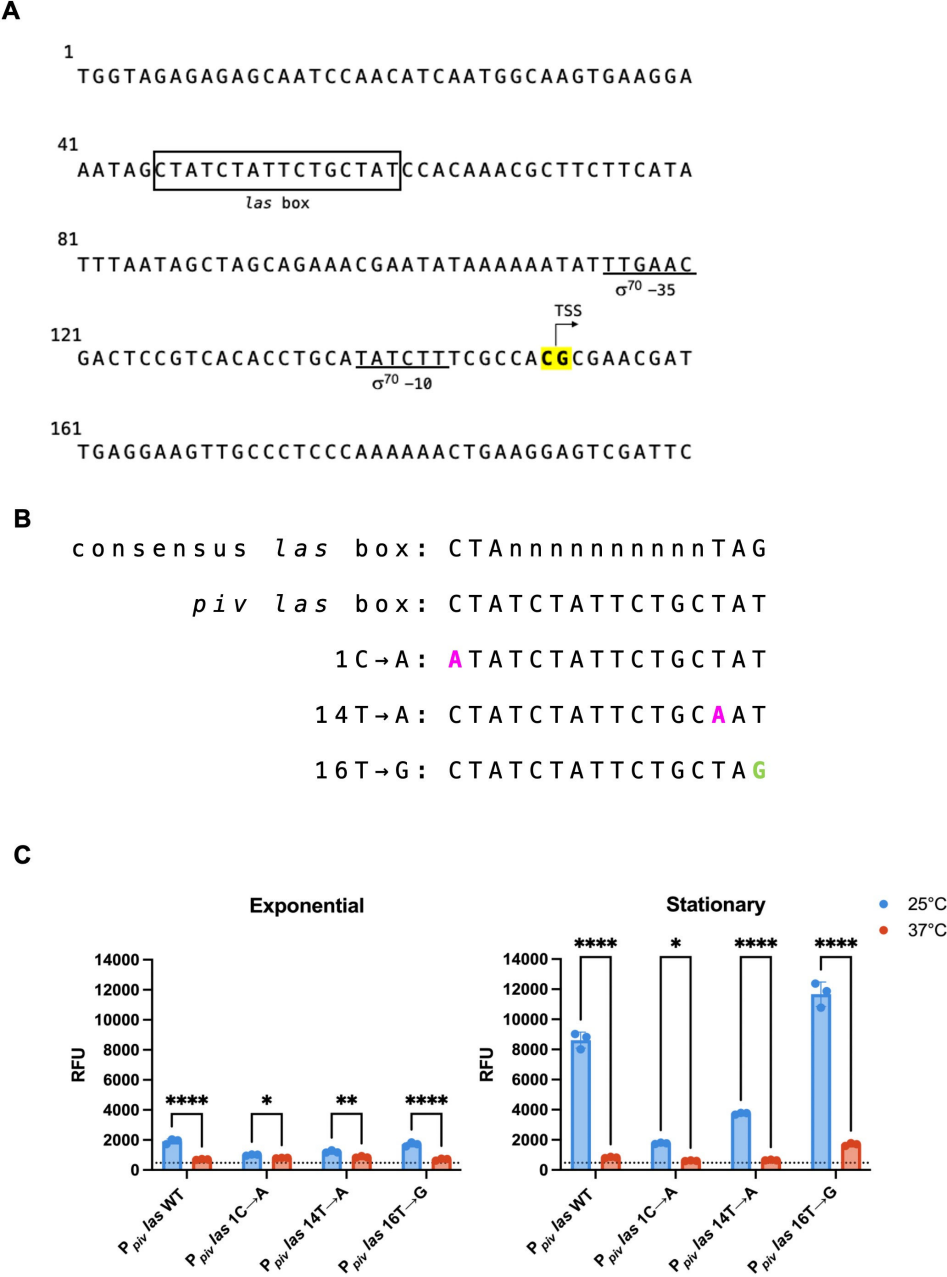
Mutations to the *piv* promoter region alter promoter activity. (**A**) Sequence of the 200 nucleotides upstream of the *piv* start codon that are sufficient for temperature and growth phase regulation. Putative σ^70^ -35 and -10 elements identified with SAPPHIRE (34) are underlined. Nearby TSSs identified by 5′ RACE are highlighted. A DNA motif with similarity to canonical *las* boxes (30) was identified upstream of transcription initiation regions and is boxed. (**B**) Mutations made to the *las* box within the *piv* promoter of the P*_piv_-gfp*(ASV) transcriptional reporter plasmid (pRD91). Mutations are predicted to disrupt (in pink) or improve (in green) LasR binding based on comparison to the consensus sequence. (**C**) PAO1 carrying *las* box mutants of the P*_piv_-gfp*(ASV) transcriptional reporter shown in panel B were grown at 25°C and 37°C, and fluorescence measurements taken at exponential (left) and stationary (right) phases as previously described. The mean of three biological replicates analyzed with standard deviation is shown. Statistical significance was determined by two-way ANOVA with Šídák multiple comparisons: *P < 0.0332, **P < 0.0021, ***P < 0.0002, and ****P < 0.0001.

To further characterize how *piv* promoter activity was thermoregulated, we analyzed the 200 nucleotides upstream of the *piv* gene more closely; this region was recently identified as containing a σ^70^-dependent promoter (28). Using SAPPHIRE (33), a predictor for σ^70^ promoters in *Pseudomonas* spp., we identified the putative -35 and -10 elements for the sigma factor RpoD (Fig. 5A, underlined regions). Putative transcription start sites (TSSs) for *piv* were mapped using 5′ rapid amplification of cDNA ends (5′ RACE) in PAO1 (Fig. 5A, highlighted bases). We found that transcription began at positions 151C or 152G of the promoter region sequence. The predicted -35 and -10 elements are appropriately positioned upstream of the TSSs such that RpoD could be directing transcription of *piv* beginning at positions 151C or 152G.

We then analyzed the region upstream of the TSSs and predicted σ^70^ −35 and −10 elements and identified a 16 bp sequence (Fig. 5A, boxed region) that resembles a canonical *las* box, particularly the one identified for non-cooperative LasR binding (29). To test this region as a putative *las* box, we introduced point mutations to the sequence in the P*_piv_-gfp*(ASV) reporter plasmid (pRD91) at highly conserved positions of a canonical *las* box sequence. If this sequence is a *las* box, mutations away from the canonical sequence should disrupt LasR binding (Fig. 5B, pink base changes), while mutations toward consensus should improve it (Fig. 5B, green base changes). The proposed deleterious mutations were selected as they have been previously shown to abolish LasR binding to and greatly reduce the activity of the *pqsR* promoter (34, 35). We then assessed the effect of these mutations on *piv* promoter activity at 25°C and 37°C in PAO1 (Fig. 5C). The predicted deleterious 1C→A and 14T→A mutations of the putative *las* box reduced *piv* promoter activity slightly at exponential phase and greatly at stationary phase at 25°C only. The 16T→G mutation, predicted to improve LasR binding, increased promoter activity at both 25°C and 37°C at stationary phase; however, promoter activity was still highly thermoregulated. The effects of these different types of mutations on *piv* promoter activity, especially at stationary phase, are consistent with the predicted effects on LasR binding and suggest that this 16 bp sequence of the promoter could be a *las* box, although we could not show that LasR directly interacts with this sequence. We have identified single nucleotides within the *piv* promoter that are crucial for the characteristic high promoter activity observed at 25°C. We have also identified a promoter mutation that results in increased activity at 37°C in an otherwise wild-type cell background.

## DISCUSSION

Here, we found that *piv* thermoregulation occurs at the level of transcriptional regulation and requires the quorum sensing regulator LasR but not the H-NS family members MvaT/MvaU. By leveraging a Δ*mvaT*Δ*mvaU* mutant strain in which *piv* expression was derepressed, we show that LasR acts as a positive regulator of *piv* at 25°C with less effect on *piv* expression at 37°C. This is consistent with our findings that *piv* expression is significantly thermoregulated at stationary phase but not at exponential phase, as LasR abundance and activity are higher at stationary phase (12). We have mapped the promoter region that contains the required *cis*-acting elements for temperature-dependent *piv* promoter activity. We also identified a putative *las* box within the *piv* promoter that could mediate direct LasR regulation and single nucleotides that are important for the wild-type *piv* promoter activity observed at 25°C and 37°C; however, we cannot exclude that LasR regulates an unidentified factor which in turn regulates *piv* more at 25°C, thus indirectly thermoregulating *piv*. While PvdS is a known regulator of *piv* in low iron conditions, since our experiments were all conducted in iron-replete lysogeny broth (LB) media in which PvdS is inactive, *piv* thermoregulation must not require PvdS. Overall, this study shows that temperature controls LasR regulation of *piv*, whether directly or indirectly, and this regulation results in higher *piv* expression at lower temperatures.

### Why is the virulence factor *piv* upregulated at low temperatures?

As PIV contributes to *P. aeruginosa* virulence in lung and burn wound infection models and is particularly crucial for the bacterium to cause corneal infections, it is perhaps surprising for *piv* to be expressed more at 25°C than the temperature physiologically relevant to humans, 37°C (17, 21, 22, 36). Many virulence factors of *P. aeruginosa* and other bacterial pathogens are upregulated in response to human body temperature, making *piv* an example of a virulence factor that is instead upregulated in response to ambient temperatures (1, 6, 7). It is important to consider that *P. aeruginosa* is an opportunistic pathogen of humans that is not only capable of infecting other organisms, such as plants and invertebrates, but also has a long evolutionary history of surviving in diverse environments as a free-living bacterium. PIV is a serine protease with a broad range of substrates that happen to include immunologically relevant molecules in humans, and its secretion likely helps the bacterium acquire nutrients and survive in a multitude of challenging environments, not specifically the human body. Presumably, *piv* is regulated by low temperature in some environments where this is beneficial for survival. That its thermoregulation is not “optimized” for human infection temperature does not belie the role of PIV in *P. aeruginosa* pathogenesis, as the iron-dependent alternate sigma factor PvdS upregulates *piv* under low iron conditions, such as those encountered in the human body. Thus, *piv* expression is affected by multiple types of environmental signals, and this complex regulation may result in a highly tunable system for controlling the production of PIV under different growth conditions.

### A model for thermoregulation of *piv* by LasR

**Fig 6:**
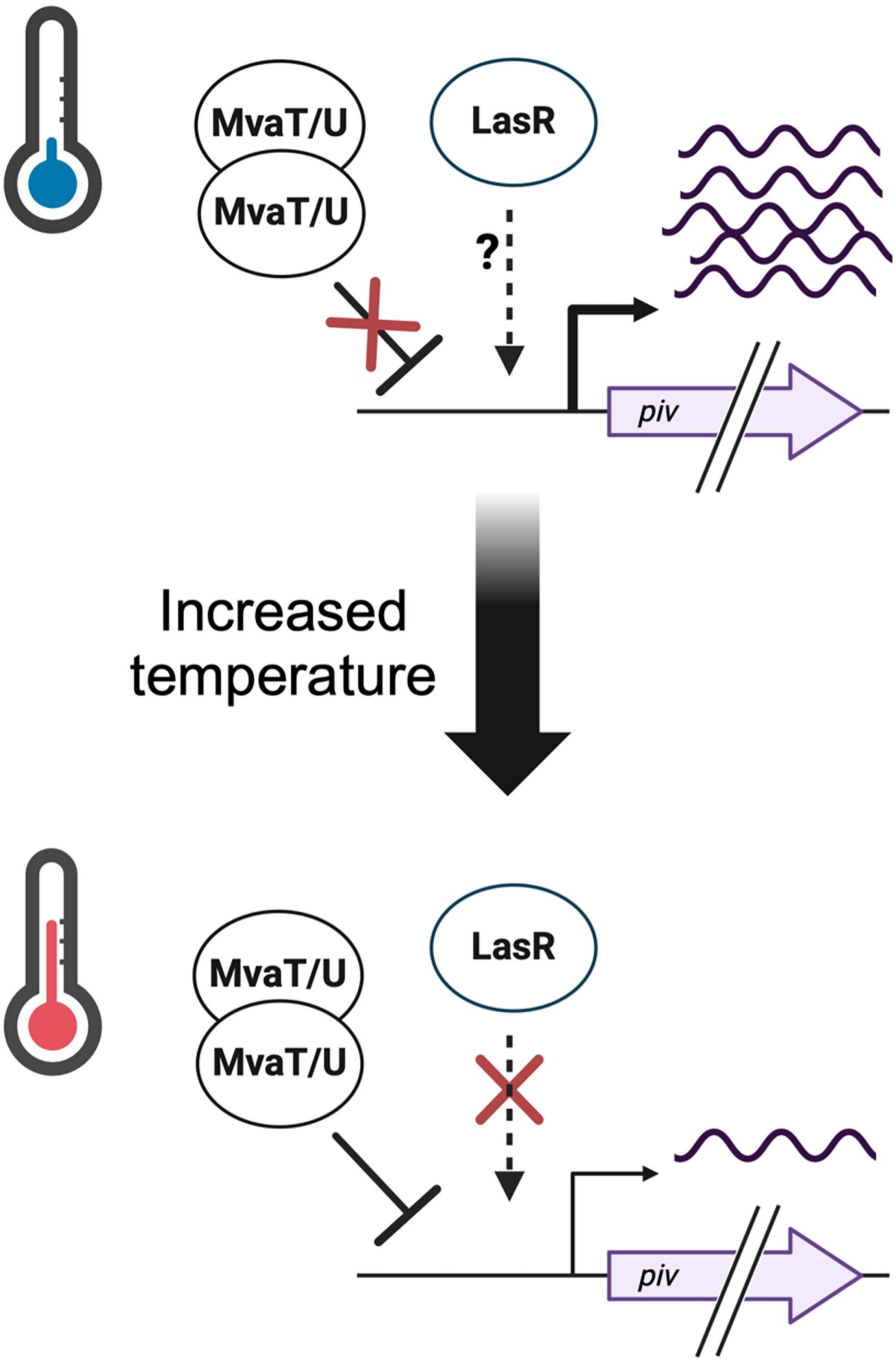
A proposed model for the transcriptional thermoregulation of *piv* by the quorum sensing regulator LasR. At 25°C (blue thermometer), LasR drives high expression of *piv* at stationary phase, either directly or indirectly via an unknown mechanism (represented by ?), while MvaT/MvaU do not repress expression. At 37°C (red thermometer), LasR no longer upregulates *piv*, while MvaT/MvaU now repress *piv* expression, leading to lower transcript levels than at 25°C. Created with BioRender.com.

We propose a model for *piv* thermoregulation in which LasR promotes *piv* expression at 25°C but not at all, or at very low levels, at 37°C and higher (Fig. 6). Although we provide compelling evidence that LasR upregulates *piv* at 25°C but not at 37°C, it remains unclear exactly how temperature is modulating LasR regulation of *piv*. In agreement with prior studies (32), we determined that LasR expression, protein, and 3O-C12-HSL levels are not higher at 25°C than at 37°C (Fig. 3). Multiple published transcriptomic studies of *P. aeruginosa* at stationary phase have also noted that the majority of LasR-regulated genes that are thermoregulated are actually upregulated at 37°C compared to a lower ambient temperature (6, 7). Thus, we do not believe that the activity of the LasRI quorum system itself is intrinsically higher at 25°C as a mechanism for thermoregulating *piv*. A second quorum sensing system activated by the LasRI system, the RhlRI system, is thermoregulated due to an RNA thermometer that results in higher RhlR levels at 37°C (32); however, *piv* is not regulated by the RhlRI quorum sensing system, and furthermore is upregulated at low temperatures, indicating that RhlRI is not responsible for *piv* thermoregulation (12). The mechanism by which low temperature promotes LasR regulation of *piv* must thus be relatively specific to the *piv* locus and not affect the entire LasR regulon. As the mechanism by which temperature regulates *piv* is inextricably linked to how LasR regulates *piv*, we believe growth temperature will be instrumental for future studies to better characterize how LasR regulates *piv*.

We postulate two possible mechanisms for how temperature affects LasR regulation of *piv*.

- LasR directly regulates *piv* and this interaction is favored at lower temperatures, leading to higher gene expression. Although we do not show direct LasR binding, mutations to the putative *las* box that would theoretically disrupt or improve LasR binding do have the expected negative or positive effects on *piv* promoter activity, consistent with direct LasR regulation. The effects are also greater at stationary phase than exponential phase. Of note is that the nucleotide at position 16 of the putative *las* box identified in the *piv* promoter is a thymine, whereas the nucleotide at position 16 of almost all *las* boxes is a guanine (7, 12, 29). Constructing the 16T→G mutation, predicted to improve LasR binding, on the putative *las* box results in increased *piv* promoter activity at 37°C (as well as increased activity at 25°C), where normally none is detected. One explanation for this is that LasR binding to the putative box is relatively poor at 37°C and lower temperatures somehow improve or favor binding. This could be due to an unidentified *trans*-acting factor present or active only at 25°C that assists in LasR binding or a *cis*-acting factor such as a “DNA thermometer,” a temperature-sensitive secondary structure that can mediate temperature-dependent transcription factor binding and subsequent gene expression (1). Poor LasR binding to the *piv* promoter at 37°C may explain why a previous study conducted at 37°C that identified promoters directly bound by LasR in PAO1 failed to identify the *piv* promoter as one, despite identifying several known and novel LasR targets (29).
- LasR indirectly regulates *piv*. Under this mechanism, LasR activates an unidentified *trans*-acting factor, which in turn positively regulates *piv* more at 25°C than at 37°C. Production and/or activity of this unidentified factor could be higher at 25°C to facilitate increased expression of *piv* at low temperatures, or regulation of *piv* itself by the factor could be favored at 25°C due to temperature-sensitive interactions with the *piv* promoter. We highlight the importance and involvement of the *piv* promoter even in an indirect mechanism, as we found the 200 nucleotides upstream of the *piv* coding sequence contain the required promoter elements for wild-type thermoregulation of *piv* promoter activity.

As previously discussed, LasR-regulated genes have been extensively studied as part of the quorum sensing regulon in *P. aeruginosa* grown at 37°C in standard laboratory conditions (10–13). Although many LasR-regulated genes have been shown to also be thermoregulated (6, 7), to the best of our knowledge, *piv* is the only gene shown to be regulated by LasR in a temperature-dependent manner. This raises the possibility that LasR could activate transcription of yet-to-be-identified genes only under specific environmental and nutritional conditions. Studying gene expression under non-standard laboratory conditions that mimic other natural and infection environments may expand our understanding of quorum sensing and virulence factor regulation in the diverse environments in which *P. aeruginosa* can survive.

## MATERIALS AND METHODS

### Bacterial strains and plasmids

Bacterial strains, plasmids, and oligonucleotides are listed in Tables S1 to S3. Standard molecular biology practices were used to construct plasmids, and all constructed plasmids were confirmed by sequencing. Mutant strains were confirmed by PCR and sequencing. Full plasmid and strain construction details are available in File S1.

### Culture conditions

Overnight cultures of *P. aeruginosa* were routinely grown in 3 mL of lysogeny broth at 37°C in a rolling drum. As appropriate, *Escherichia coli* growth media were supplemented with gentamicin (15 µg/mL), carbenicillin (100 µg/mL), or tetracycline (10 µg/mL) and *P. aeruginosa* media with gentamicin (60 µg/mL), carbenicillin (300 µg/mL), or tetracycline (100 µg/mL), unless otherwise noted. 3O-C12-HSL was obtained commercially (Sigma-Aldrich) and dissolved in 0.01% acidified ethyl acetate. In all experiments with HSLs, HSLs were added to a glass culture vessel several hours prior to the addition of bacteria, and the ethyl acetate was allowed to evaporate.

### RNA extraction and DNase treatment

To measure transcript levels at different growth temperatures, biological triplicates of PAO1 grown overnight at 37°C were subcultured to an initial OD_600_ of 0.05 in 25 mL LB and incubated, shaking at 200 rpm at the indicated temperature. At exponential phase (OD_600_ ~ 0.5), 10^9^ cells were harvested, resuspended in 1 mL Tri-Reagent (Millipore Sigma), and RNA was extracted according to the manufacturer’s instructions, using chloroform. The cultures were allowed to continue growing until early stationary phase (OD_600_ ~ 2.0), and RNA was extracted again as described. Extracted RNA (5 µg) was DNase treated with TURBO DNase (ThermoFisher) according to the manufacturer’s instructions for rigorous DNase treatment.

### Real-time quantitative PCR

One-step RT-qPCR was performed on DNase-treated RNA using Power SYBR Green RNA-to-CT 1-Step Kit (Applied Biosystems) on a LightCycler 96 (Roche) using LightCycler software version 1.1.0.1320. Reactions were conducted in technical triplicate for each biological sample, and the *C*_*q*_ values were averaged for gene expression analysis using the temperature-insensitive *omlA* (6) as an internal control gene. The relative expression of a gene at a given temperature *X*°C compared to 37°C was calculated and plotted as log_2_(2^-ΔΔCt^), where ΔΔCt = (*C*_*q*_ gene at *X*°C − *C*_*q*_ *omlA* at *X*°C) – (*C*_*q*_ gene at 37°C – *C*_*q*_ *omlA* at 37°C).

### Immunoblotting

Overnight biological triplicates were subcultured to an initial OD_600_ of 0.05 in 25 mL LB and incubated shaking at 200 rpm at the indicated temperature. At early stationary (OD_600_ ~ 2.0) phase, 0.5 mL was harvested and resuspended in 475 µL Laemmli buffer with 25 µL β-mercaptoethanol and boiled for 15 minutes. A volume of 10 µL of prepared cell lysate sample was electrophoresed, transferred to a low-fluorescence PVDF membrane (Bio-Rad), and blocked for 1 hour in Intercept Blocking Buffer (Li-Cor). Primary antibodies against the VSV-G epitope tag (Sigma, batch # 0000143676) were used at 1:5,000 overnight at 4°C and primary antibodies against LasR (12) were used at 1:1,000 overnight at 4°C with fluorophore-conjugated secondary antibodies against rabbits used at 1:10,000 (Li-Cor, Lot No. D20803-09). Primary antibodies against the α-subunit of RNA polymerase were used at 1:5,000 (BioLegend, Lot No. B376827) with fluorophore-conjugated secondary antibodies against mice (Li-Cor, Lot No. D20601-01) used at 1:10,000. Membranes were imaged on a BioRad ChemiDoc Imager. Primary antibodies against LasR were validated by immunoblotting cell lysate from PAO1 and PAO1 Δ*lasR* (Fig. S3). Densitometry analysis was conducted on raw image files using ImageJ.

### 3O-C12-HSL quantification

Overnight biological triplicates of PAO1 were subcultured to an initial OD_600_ of 0.05 and incubated at 25°C and 37°C, shaking at 200 rpm until early stationary phase (OD_600_ ~ 2.0). HSLs were extracted from 2 mL of culture with 0.01% acidified ethyl acetate as previously described (37). To measure 3O-C12-HSL concentrations, a bioreporter strain of *E. coli* that produces GFP in response to 3O-C12-HSL was used as described with minor modifications (38–40). Briefly, an overnight culture of the bioreporter *E. coli* carrying pJNL and pPROBE_rsal_ was subcultured 1:100 in LB containing the appropriate antibiotics and grown for 2.5 hours before induction with 0.4% L-arabinose for an additional 30 minutes. After induction, 500 µL of culture was added to glass culture tubes containing the HSLs extracted from PAO1 and incubated at 37°C for 1 hour. A volume of 100 µL of each culture was transferred to a black-sided 96-well plate, and fluorescence was measured (excitation 485 nm, emission 515 nm, and gain 100) on a Synergy H1 plate reader (BioTek). A linear standard curve of commercial 3O-C12-HSL was used to determine the concentration of 3O-C12-HSL in samples.

### Transcriptional reporter assays

Biological triplicates of strains with a transcriptional reporter plasmid were grown overnight at 37°C and subcultured to an initial OD_600_ of 0.1 in 25 mL LB supplemented with gentamicin 30 µg/mL and incubated, shaking at 200 rpm at both 25°C and 37°C. At exponential phase (OD_600_ ~ 0.5), 10^9^ cells were harvested at 9,000 × *g* for 2 minutes, supernatant removed, and the pellet resuspended in 1 mL PBS. A volume of 200 µL of each sample was then added to a black-sided 96-well plate in technical triplicate, and the fluorescence was measured (excitation 485 nm, emission 515 nm, and gain 100) on a Synergy H1 plate reader (BioTek). At stationary phase (OD_600_ ~ 2.0), 10^9^ cells were sampled from the same cultures, and fluorescence was measured as described. The RFU of 200 µL PBS on each plate was subtracted from technical replicates, which were then averaged per biological replicate. Background was determined by averaging all values from a transcriptional reporter assay of PAO1 carrying pRD87, a no-promoter control of the reporter plasmid.

### 5′ rapid amplification of cDNA ends

PAO1 was grown at 25°C in LB to the late log phase (OD_600_ ~ 0.8). RNA was extracted using the MasterPure RNA Purification Kit (Epicentre) and treated with TURBO DNase as described. 5′ RACE was conducted using the 5′ RACE System for Rapid Amplification of cDNA Ends (ThermoFisher) according to the manufacturer’s instructions with modifications: ThermoScript Reverse Transcriptase (Invitrogen) was used in place of the kit’s reverse transcriptase with betaine added to 0.5 M for the reverse transcriptase step.

### Statistical analyses

All statistical tests were conducted as described using GraphPad Prism version 10.

## SUPPLEMENTAL MATERIAL

Supplemental Material mbio.00541-25-s0001.pdf Supplemental text, figures, and tables.

ASM does not own the copyrights to Supplemental Material that may be linked to, or accessed through, an article. The authors have granted ASM a non-exclusive, world-wide license to publish the Supplemental Material files. Please contact the corresponding author directly for reuse.
