# Supplementary material for: Temperature controls LasR regulation of piv expression in Pseudomonas aeruginosa
Source: mBio. 2025 May 20;16(6):e00541-25. doi: 10.1128/mbio.00541-25 (PMC12153295; doi:10.1128/mbio.00541-25)
Supplement: Supplemental Material — Supplemental text, figures, and tables. [file mbio.00541-25-s0001.pdf]

SUPPLEMENTAL MATERIALS

Temperature controls LasR regulation of *piv* expression in *Pseudomonas aeruginosa*

Rachel E. Robinson[a,b], Joshua K. Robertson[c], Samantha M. Prezioso[a,b*], and Joanna B. Goldberg[b,d#]

[a]Microbiology and Molecular Genetics Program, Graduate Division of Biological and Biomedical Sciences, Laney Graduate School, Emory University, Atlanta, Georgia, USA

[b]Department of Pediatrics, Division of Pulmonary, Asthma, Cystic Fibrosis, and Sleep, Emory University School of Medicine, Atlanta, Georgia, USA

[c]Department of Biology, Emory University, Atlanta, Georgia, USA

[d]Emory+Children's Center for Cystic Fibrosis and Airway Disease Research, Emory University School of Medicine, Atlanta, Georgia, USA

*Present address: Alltrna, Boston, Massachusetts, USA

Supplemental Methods

Plasmid construction

All plasmids were constructed by isothermal assembly (1) using Gibson Assembly Master Mix (New England Biolabs [NEB], Ipswich, MA, USA) according to manufacturer's protocol prior to transformation by heat shock into competent *Escherichia coli* 5-alpha (NEB) also according to the manufacturer's protocol. Plasmids were sequenced by Genewiz/Azenta (Azenta Life Sciences, Chelmsford, MA, USA) or Plasmidsaurus (Eugene, OR, USA) to confirm the assembled product. For construction of the following plasmids, *Pseudomonas aeruginosa* PAO1 genomic DNA (gDNA) was extracted using the Qiagen DNeasy Blood & Tissue Kit according to the manufacturer's recommendations, used as template in PCR reactions with the indicated primers unless otherwise noted, and then assembled into the indicated plasmid backbone that had been previously digested with the indicated restriction enzyme(s).

Plasmid pEXG2 is an allelic exchange vector that enables the construction of chromosomal mutations in PAO1 and derived strains (2). To construct pEXG2_*piv-vsvG*, the PCR product of oRD208 and oRD209, the latter of which added the sequence for the vesicular stomatitis virus G epitope tag (VSV-G) to the C-terminus of the *piv* coding region, was then PCR amplified with oRD208 and oRD210 and assembled with the PCR product of oRD211 and oRD212 into pEXG2 digested with XbaI and HindIII (NEB). Plasmid pEXG_*lasR*_del facilitated deletion of amino acids 2-237 from the *lasR* coding region in order to not disrupt the overlapping coding region for *rsaL*. pEXG_*lasR*_del was construct by assemble of the PCR product of oRD89 and oRD90 with the PCR product of oRD91 and oRD92 into pEXG2 digested with HindIII.

Plasmids pACRISPR and pCasPA form a two plasmid system for genomic editing in *P. aeruginosa* via CRISPR-Cas9 activity followed by λ-Red recombination (3). For the following plasmids, pACRISPR was engineered with the desired spacer to direct Cas9 cleavage as well as with repair DNA required for λ-Red recombination to repair the DNA break as previously described (3). Briefly, spacers for *lasR* composed of annealed oRD113/oRD114 and *lasI* composed of annealed oRD177/oRD178 were inserted into BsaI-digested pACRISPR by Golden Gate assembly (4). For pACRISPR_*lasR* construction, PCR products of oRD115 and oRD116 were assembled with PCR products of oRD117 and oRD118 into the respective spacer containing intermediate pACRISPR plasmid digested with XbaI and XhoI (NEB). For pACRISPR_*lasI*, PCR products of oRD179 and oRD180 and PCR products of oRD181 and oRD182 were assembled into the respective spacer intermediate pACRISPR plasmid in the exact same manner.

Plasmid pUCP18T-mini-Tn*7*T-Gm was used for chromosomal insertions at the neutral *att*Tn*7* site (5). For the following derived plasmids, PCR products were assembled into pUCP18T-mini-Tn*7*T-Gm digested with BamHI and HindIII (NEB). PCR products of oRD170 and oRD171, of oRD166 and oRD167, and of oRD168 and oRD169 were used to construct pUC18T-mini-Tn*7*T-Gm_P_{lasR}-*lasR*, pUC18T-mini-Tn*7*T-Gm_P_{mvaT}-*mvaT*, and pUC18T-mini-Tn*7*T-Gm_P_{mvaU}-*mvaU*, respectively.

Plasmid pUCP30T was used as an empty vector for transcriptional reporter assays, with modifications to the backbone as follows: the *lac* operon was removed from pUCP30T by site directed mutagenesis to remove transcriptional readthrough. pUCP30T was PCR amplified with oRD218 and pRD219 and the resulting product digested with DpnI, phosphorylated with polynucleotide kinase, and ligated with T4 DNA ligase, all according to the manufacturer's instructions (NEB). The resulting pUCP30T Δ*lac* plasmid was digested with HindIII and assembled with PCR products of oRD65 and oRD66 and PCR products of oRD67 and oRD68 off

pCdrA::*gfp*(ASV)^{C} to create pUCP30T Δ*lac* operon_P_{piv}-*gfp(*ASV). Site directed mutagenesis of pUCP30T Δ*lac* operon_P_{piv}-*gfp(*ASV) with oRD213 and oRD217 was used to remove the *piv* promoter and construct the no-promoter pUCP30T Δ*lac* operon_*gfp*(ASV). Site directed mutagenesis of pUCP30T Δ*lac* operon_P_{piv}-*gfp(*ASV) with oRD155 and either oRD160, oRD163, or oRD157 was used to construct pUCP30T Δ*lac* operon_P_{piv}-*las*$^{1C\text{-}A}$-*gfp(*ASV), pUCP30T Δ*lac* operon_P_{piv}-*las*$^{14T\text{-}A}$-*gfp(*ASV), or pUCP30T Δ*lac* operon_P_{piv}-*las*$^{16T\text{-}G}$-*gfp(*ASV), respectively.

Transformation of strains

P. aeruginosa strains were transformed by electroporation. Briefly, overnight cultures of *P. aeruginosa* were centrifuged, washed twice with 300 mM sucrose, and 100 μL of 200 μL resuspended cells electroporated at 2500V with plasmid(s). Cells were immediately recovered in 1 mL SOC rolling at 37°C for an hour and plated on appropriate antibiotic media overnight at 37°C.

E. coli SM10 grown to ~OD_{600} 0.6-0.8 were chilled on ice for 10 minutes and washed twice with cold 0.1M $CaCl_2$ containing 15% glycerol before transformation by standard heat shock and recovery and plating as for *P. aeruginosa* transformations.

Specialized media

For the below conjugations, VBMM media was made by autoclaving 15 g agar in 900 mL water and adding 100 mL of 10X sterile VBMM stock solution (30 g/L trisodium citrate, 20 g/L citric acid, 100 g/L K_2HPO_4, 35 g/L $NaNH_4PO_4 \bullet 4H_2O$), 1 mL of 1 M $MgSO_4$, and 0.1 mL of 1 M $CaCl_2$. No salt LA (NSLA) with 15% sucrose was made on the same day as used by autoclaving 10 g tryptone, 5 g yeast extract, and 15 g agar in 700 mL water and adding 300 mL of freshly made 50% sterile sucrose.

Strain construction

To create the PAO1 *piv-vsvG* strain with in-frame C-terminal VSV-G tagged *piv* at the native chromosomal locus, PAO1 was conjugated with *E. coli* SM10 carrying plasmid pEXG2_*piv-vsvG* using an allelic exchange method (2) with modifications as follows. An overnight culture of PAO1 grown at 37°C was supplemented with 3 mL additional LB and then incubated at 42°C. At the same time, an overnight of *E. coli* SM10 pEXG2_*piv-vsvG* was subcultured 1:100 in 10 mL of LB with gentamicin. After three hours, 500 μL of PAO1 and 1.5 mL of donor *E. coli* were centrifuged, resuspended together in 50 μL LB, and puddle mated on an LB agar overnight at 30°C. Puddles were resuspended in 1 mL phosphate buffered saline (PBS) and plated on VBMM containing gentamicin overnight at 37°C. A single colony was restreaked on LA with gentamicin and incubated overnight at 37°C. A single colony was used to inoculate 1 mL LB and incubated in the rolling drum at 37°C for approximately 3.5 hours, or until growth in liquid was just visible, and dilutions plated on freshly made NSLA 15% sucrose media. Resulting colonies were screened for growth on media containing sucrose but not the antibiotic and confirmed by PCR and sequencing of the region of interest. The Δ*mvaT*Δ*mvaU*Δ*lasR* mutant strain was constructed by conjugating Δ*mvaT*Δ*mvaU* with *E. coli* SM10 carrying plasmid pEXG2_*lasR*_del as described. Notably, *lasR* could only be deleted from Δ*mvaT*Δ*mvaU* by allelic exchange and not by CRISPR-Cas9 genome editing as described in the next paragraph.

PAO1 Δ*lasR* and PAO1 Δ*lasI* mutants were constructed using a CRISPR-Cas9 system to create deletions (3) with minor modifications to the transformation protocols specifically. For PAO1 Δ*lasR*, the sequence encoding amino acids 237-240 was not deleted in order to retain the entire sequence of the overlapping *rsaL*. PAO1 was first transformed with pCasPA by electroporation. An overnight culture of the resulting PAO1 pCasPA strain was subcultured 1:20 in 10 mL LB with

no antibiotic for 3 hours at 37°C. Arabinose was added to a final concentration of 2 mg/mL. After two hours, cells were centrifuged and electroporated with pACRISPR_*lasR* to create Δ*lasR*, and pACRISPR_*lasI* to create Δ*lasI*. Single colonies were passed on NSLA 15% sucrose media to cure plasmids and screened by PCR and sequencing to confirm the desired deletion.

Mutant strains were complemented by transformation of a pUC18T-mini-Tn*7*T-Gm derived plasmid and the pTNS3 transposase helper plasmid (5). The gentamicin marker was removed from transformants using the pFLP2 Flp recombinase containing plasmid, and pFLP2 cured from strains by passing on NSLA 15% sucrose media. pUC18T-mini-Tn7T-Gm_P_{lasR}-*lasR* was used to make PAO1 Δ*lasR* Tn*7*:: P_{lasR}-*lasR,* pUC18T-mini-Tn*7*T-Gm_P_{mvaT}-*mvaT* to make PAO1 Δ*mvaT*Δ*mvaU* Tn*7*:: P_{mvaT}-*mvaT*, and pUC18T-mini-Tn*7*T-Gm_P_{mvaU}-*mvaU* to make PAO1 Δ*mvaT*Δ*mvaU* Tn*7*:: P_{mvaU}-*mvaU*.

Table S1. Bacterial strains used in this study.

Strain	Source
Escherichia coli 5-alpha (DH5α derivative)	New England Biolabs (NEB)
Escherichia coli SM10	This study
Pseudomonas aeruginosa PAO1	Simon Dove (Harvard University)
PAO1 *mvaT-vsvG*	Lippa et al., 2020 (6)
PAO1 *mvaU-vsvG*	Lippa et al., 2020 (6)
PAO1 *piv-vsvG*	This study
PAO1 Δ*lasR*	This study
PAO1 Δ*lasI*	This study
PAO1 Δ*mvaT*Δ*mvaU*	Simon Dove (Harvard University)
PAO1 Δ*mvaT*Δ*mvaU*Δ*lasR*	This study
PAO1 Δ*lasR* Tn*7*:: P_{lasR}-*lasR*	This study
PAO1 Δ*mvaT*Δ*mvaU* Tn*7*:: P_{mvaT}-*mvaT*	This study
PAO1 Δ*mvaT*Δ*mvaU* Tn*7*:: P_{mvaU}-*mvaU*	This study

Table S2. Plasmids used in this study.

Short Name	Plasmid	Description	Source
pRD50	pEXG2	Empty allelic exchange vector	Hmelo et al., 2015 (2)
pRD86	pEXG2_*piv-vsvG*	Introduction of *piv-vsvG* at native chromosomal locus	This study
pRD62	pEXG2_*lasR*_del	Deletion of *lasR* through amino acid 236/240	This study
pRD74	pCasPA	Express Cas9 nuclease and λ-Red system	Chen et al., 2018 (3)
pRD73	pACRISPR	Empty vector for sgRNA expression	Chen et al., 2018 (3)
pRD77	pACRISPR_*lasR*	Deletion of *lasR* through amino acid 236/240	This study
pRD84	pACRISPR_*lasI*	Deletion of *lasI*	This study
pRD16	pUC18T-mini-*Tn7*T-Gm	Empty vector for chromosomal insertion at *att*Tn*7* site	Choi and Schweizer, 2006 (5)
pRD34	pTNS3	Helper plasmid for inserting Tn*7* constructs at *att*Tn*7* site	Choi and Schweizer, 2006 (5)
pRD63	pFLP2	Flp recombinase to remove Gm marker from chromosomal *att*Tn*7* insertions	Choi and Schweizer, 2006 (5)
pRD82	pUC18T-mini-Tn*7*T-Gm_P_{lasR}-*lasR*	Complementation of PAO1 Δ*lasR*	This study
pRD80	pUC18T-mini-Tn*7*T-Gm_P_{mvaT}-*mvaT*	Complementation of PAO1 Δ*mvaT*Δ*mvaU* with only MvaT	This study
pRD81	pUC18T-mini-Tn*7*T-Gm_P_{mvaU}-*mvaU*	Complementation of PAO1 Δ*mvaT*Δ*mvaU* with only MvaU	This study
pRD54	pCdrA::*gfp*(ASV)[C]	Source of *gfp*(ASV)	Rybtke et al., 2012 (7)

n/a	pJNL	Plasmid to express LasR in bioreporter *E. coli* system	Wellington and Greenberg, 2019 (8)
n/a	pPROBE$_{rsaL}$	GFP-based reporter for LasR activity in bioreporter *E. coli* system	Wellington and Greenberg, 2019 (8)
pRD51	pUCP30T	Empty vector for transcriptional reporters	HP Schweizer
pRD87	pUCP30T Δ*lac* operon_*gfp*(ASV)	No promoter *gfp*(ASV) transcriptional reporter	This study
pRD91	pUCP30T Δ*lac* operon_P$_{piv}$-*gfp*(ASV)	Transcriptional reporter with *gfp*(ASV) under control of *piv* promoter	This study
pRD94	pUCP30T Δ*lac* operon_P$_{piv}$-*las*$^{1C\text{-}A}$-*gfp*(ASV)	Transcriptional reporter with *gfp*(ASV) under control of *piv* promoter with *las* box mutation 1C→A	This study
pRD95	pUCP30T Δ*lac* operon_P$_{piv}$-*las*$^{14T\text{-}A}$-*gfp*(ASV)	Transcriptional reporter with *gfp*(ASV) under control of *piv* promoter with *las* box mutation 14T→A	This study
pRD98	pUCP30T Δ*lac* operon_P$_{piv}$-*las*$^{16T\text{-}G}$-*gfp*(ASV)	Transcriptional reporter with *gfp*(ASV) under control of *piv* promoter with *las* box mutation 16T→G	This study

Table S3. Primers used in this study.

Primer	Sequence	Description	Source
oRD15	CCTGCTGAACAACGGCAAC	qPCR *piv* forward	This study
oRD16	AGCACTGGGTGGTGTTGTAG	qPCR *piv* reverse	This study
oRD65	GGTACCCGGGGATCCTCTAGAGTCGACCTGCAGGCATGCAtggtagagagagcaatccaa	pRD91 construction	This study
oRD66	TTGGGACAACTCCAGTGAAAAGTTCTTCTCCTTTACGCATgaatcgactccttcagtttt	pRD91 construction	This study
oRD67	atgcgtaaaggagaagaact	pRD91 construction	This study
oRD68	TTTTCCCAGTCACGACGTTGTAAAACGACGGCCAGTGCCAattctcaccaataaaaaacg	pRD91 construction	This study
oRD73	AAAATCGACATCCAGCAAGG	qPCR *omlA* forward	This study
oRD74	GGTCGCTGTCGTTGAAGAAC	qPCR *omlA* reverse	This study
oRD89	CACATTATACGAGCCGGAAGCATAAATGTAAAGCAAGCTTccgatgacgccggcgcagat	pRD62 construction	This study
oRD90	CGCCAGCTCGCCGACCTGAGAGGCAAGATCAGAGAGTAATcatagcgctacgttcttctt	pRD62 construction	This study
oRD91	attactctctgatcttgcct	pRD62 construction	This study
oRD92	TCGAGCCCGGGGATCCTCTAGAGTCGACCTGCAGAAGCTTcgagccgacaggtccccgtc	pRD62 construction	This study
oRD113	GTGGgctcaagtggaaaattggag	pRD77 construction	This study
oRD114	AAACctccaattttccacttgagc	pRD77 construction	This study
oRD115	CGAGTCGGTGCTTTTTTTGAGATCTGTCCATACCCATGGTccgaactggaaaagtggc	pRD77 construction	This study
oRD116	tcgccagctcgccgacctgagaggcaagatcagagagtaaagcgctacgttcttcttaaac	pRD77 construction	This study
oRD117	ttactctctgatcttgcctctc	pRD77 construction	This study
oRD118	AGAATACTCAAGCTTCTGAATGGCGGGAGTATGAAAAGTCcttcaggagtatcttcctgg	pRD77 construction	This study
oRD155	ATTTCCTTCACTTGCCAT	pRD91 SDM, universal reverse	This study
oRD157	AGCTATCTATTCTGCTAGCC	pRD91 SDM, *las* box 16T-G	This study
oRD160	AGATATCTATTCTGCTATCC	pRD91 SDM, *las* box 1C-A	This study
oRD163	AGCTATCTATTCTGCAATCC	pRD91 SDM, *las* box 14T-A	This study
oRD166	gcttttgaagctaattcgatcatgcatgagctcactagtggggtatactccagaaacatcgcgaaagg	pRD80 construction	This study
oRD167	tctggttggcctgcaaggccttcgcgaggtaccgggcccattagccgagcagggtggc	pRD80 construction	This study
oRD168	gcttttgaagctaattcgatcatgcatgagctcactagtgttcgcccgactctcccctgg	pRD81 construction	This study
oRD169	tctggttggcctgcaaggccttcgcgaggtaccgggcccattagcgttgcagccaggattcg	pRD81 construction	This study
oRD170	gcttttgaagctaattcgatcatgcatgagctcactagtggctgtgcctttgcgcgt	pRD82 construction	This study

oRD171	tctggttggcctgcaaggccttcgcgaggtaccgggccca tcagagagtaataagacccaaattaacggcca	pRD82 construction	This study
oRD177	GTGGctctggacagaaaggctcgc	pRD84 construction	This study
oRD178	AAACgcgagcctttctgtccagag	pRD84 construction	This study
oRD179	CGAGTCGGTGCTTTTTTTGAGATCTGTC CATACCCATGGTgggagatatcggttatctgc	pRD84 construction	This study
oRD180	tccgacagagaacgcgccggcgcgagccgacaggtcc ccgcttcacttcctccaaatagg	pRD84 construction	This study
oRD181	cggggacctgtcggct	pRD84 construction	This study
oRD182	AGAATACTCAAGCTTCTGAATGGCGGGA GTATGAAAAGTCactggaacgcctcagccagg	pRD84 construction	This study
oRD189	TACCAGAAGTCCCTGCGTGA	qPCR *mvaT* forward	This study
oRD190	GCGGGTTCTTGTACTGCTTG	qPCR *mvaT* reverse	This study
oRD191	GCACAACATCATCGCCATCC	qPCR *mvaU* forward	This study
oRD192	TCGACGACTTCACCGTTGTT	qPCR *mvaU* reverse	This study
oRD199	GGCCTTGGTTGACGGTTTTC	qPCR *lasR* forward	This study
oRD200	CAACAGGCCGAACAGGATCT	qPCR *lasR* reverse	This study
oRD208	ccggaagcataaatgtaaagcaagcttctgcaggtcgac tgcgacgctgcagacca	pRD86 construction	This study
oRD209	tcattttcctaatctattcatttcaatatctgtatatgcggccgc gggcgcgaagtagcggga	pRD86 construction	This study
oRD210	gatcgatagcctggctggcctcattttcctaatctattcatttc aatatctgtatatgcgg	pRD86 construction	This study
oRD211	gccgcatatacagatattgaaatgaatagattaggaaaat gaggccagccaggctatcga	pRD86 construction	This study
oRD212	ggaaattaattaaggtaccgaattcgagctcgagcccgg gcattcctcctgcccctcc	pRD86 construction	This study
oRD213	TGCATGCCTGCAGGTCGA	pRD87 construction	This study
oRD217	ATGCGTAAAGGAGAAGAACT	pRD87 construction	This study
oRD218	caggaaacagctatgacc	pRD91 construction	This study
oRD219	gtgccagctgcattaatg	pRD91 construction	This study
pSMP171	CGCTTGGGGTTGGCGAAG	5' RACE, GSP1	This study
pSMP172	CCTGGAGCACGGCGACGT	5' RACE, GSP 2	This study
pSMP173	CGTCGCCAGCCATTTCGCT	5'RACE, nested GSP	This study

Supplemental Figures

Figure S1

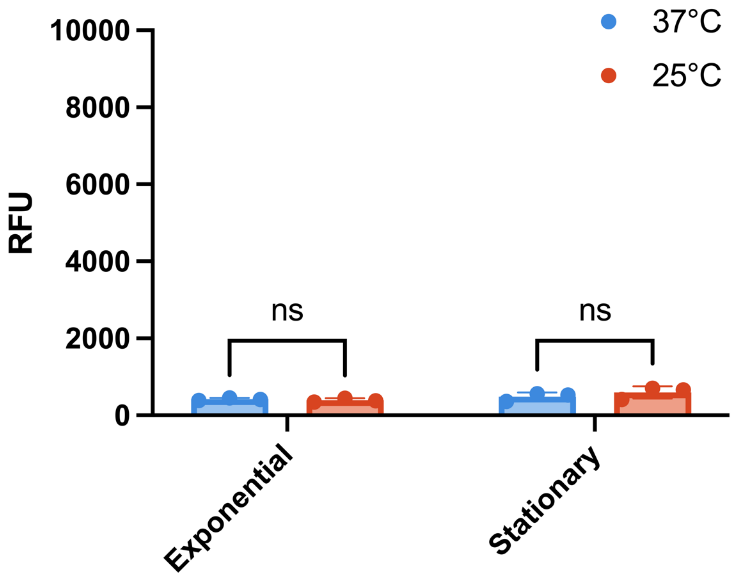

Fig. S1. PAO1 carrying a no-promoter *gfp*(ASV) reporter plasmid (pRD87) was grown at 25°C and 37°C. An equal number of cells were sampled for fluorescence (RFU) measurements (excitation 485 nm, emission 515 nm, and gain 100) first at exponential and then at early stationary phase. Three biological replicates were performed, with RFU measurements conducted in technical triplicate per each. The mean of three biological replicates is shown with error bars representing standard deviation. Statistical significance was determined by two-way repeated measures (RM) ANOVA with Šídák multiple comparisons, ns – not significant.

Figure S2

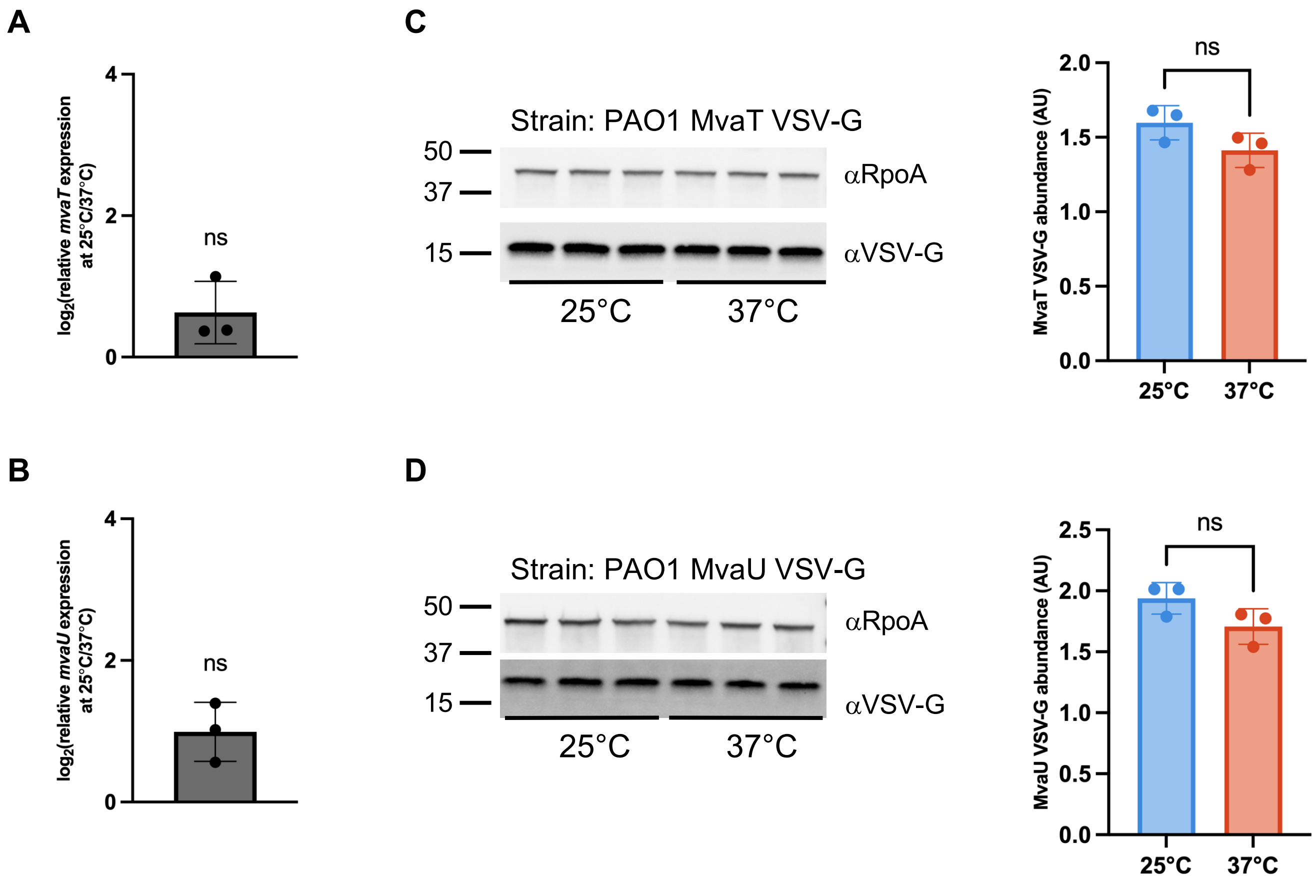

Fig. S2. A, B) PAO1 was grown at 25°C and 37°C and RNA extracted at stationary phase as previously described. Expression of *mvaT* (A) and *mvaU* (B) was determined by RT-qPCR using *omlA* as an internal control gene and calculated for 25°C relative to 37°C. RT-qPCR was conducted in technical triplicate. The mean of three biological replicates analyzed is shown with error bars representing standard deviation. Statistical significance was determined by one sample t-test with the hypothetical value = 0 for no thermoregulation, ns – not significant. C, D) Immunoblots (left) and subsequent quantification (right) of the indicated transcriptional regulator in PAO1 grown to stationary phase at 25°C and 37°C. C) PAO1 MvaT VSV-G was probed with αVSV-G antibodies. D) PAO1 MvaU VSV-G was probed with αVSV-G antibodies. All samples were probed with αRpoA as a loading control. Biological triplicates for each protein are shown. For densitometry analysis, the amount of the indicated protein was determined relative to the amount of RpoA in the same sample using ImageJ. The mean of three biological replicates is shown with standard deviation. Statistical significance was determined by two-tailed t test, ns – not significant.

Figure S3

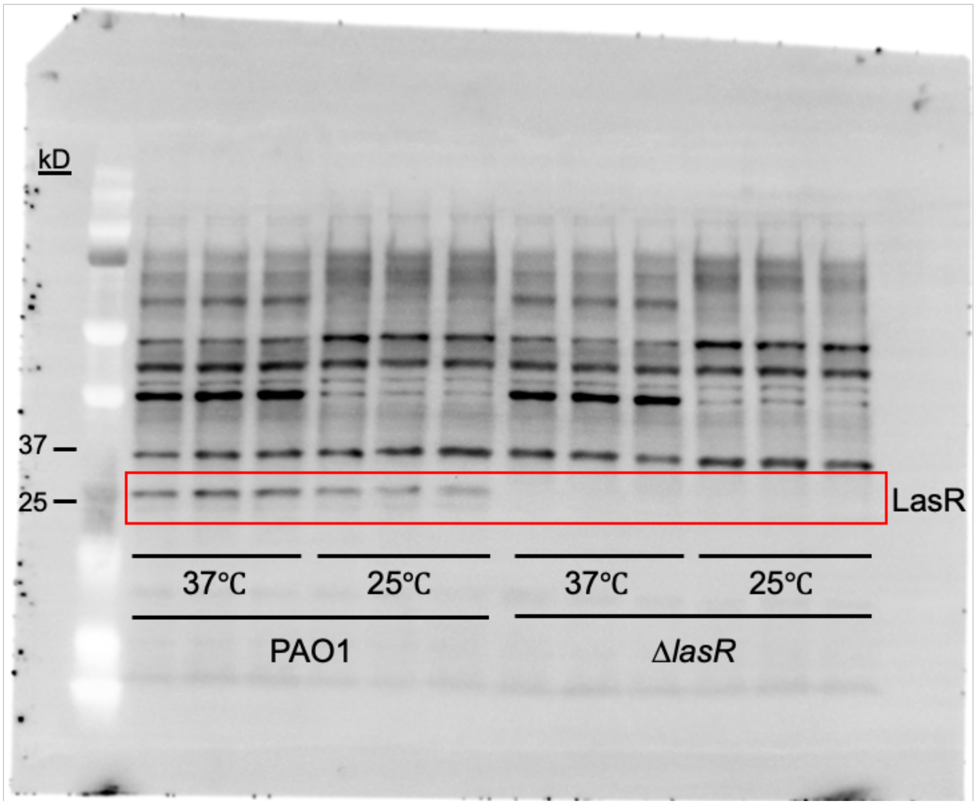

Fig. S3. Verification of αLasR antibodies to detect LasR by immunoblotting. Cell lysates from PAO1 and Δ*lasR* grown to early stationary at 25°C and 37°C were probed with αLasR antibodies. The red box indicates the position of LasR at ~26 kD, which is present in PAO1 strains at both 37°C and 25°C but not in Δ*lasR* at either temperature.

Supplemental References

1. Gibson DG, Young L, Chuang R-Y, Venter JC, Hutchison CA, Smith HO. 2009. Enzymatic assembly of DNA molecules up to several hundred kilobases. Nat Methods 6:343–345.

2. Hmelo LR, Borlee BR, Almblad H, Love ME, Randall TE, Tseng BS, Lin C, Irie Y, Storek KM, Yang JJ, Siehnel RJ, Howell PL, Singh PK, Tolker-Nielsen T, Parsek MR, Schweizer HP, Harrison JJ. 2015. Precision-engineering the Pseudomonas aeruginosa genome with two-step allelic exchange. Nat Protoc 10:1820–1841.

3. Chen W, Zhang Y, Zhang Y, Pi Y, Gu T, Song L, Wang Y, Ji Q. 2018. CRISPR/Cas9-based Genome Editing in Pseudomonas aeruginosa and Cytidine Deaminase-Mediated Base Editing in Pseudomonas Species. iScience 6:222–231.

4. Engler C, Gruetzner R, Kandzia R, Marillonnet S. 2009. Golden Gate Shuffling: A One-Pot DNA Shuffling Method Based on Type IIs Restriction Enzymes. PLOS ONE 4:e5553.

5. Choi K-H, Schweizer HP. 2006. mini-Tn 7 insertion in bacteria with single att Tn 7 sites: example Pseudomonas aeruginosa. Nature Protocols 1:153–161.

6. Lippa AM, Gebhardt MJ, Dove SL. 2021. H-NS-like proteins in Pseudomonas aeruginosa coordinately silence intragenic transcription. Mol Microbiol 115:1138–1151.

7. Rybtke MT, Borlee BR, Murakami K, Irie Y, Hentzer M, Nielsen TE, Givskov M, Parsek MR, Tolker-Nielsen T. 2012. Fluorescence-Based Reporter for Gauging Cyclic Di-GMP Levels in Pseudomonas aeruginosa. Applied and Environmental Microbiology 78:5060–5069.

8. Wellington S, Greenberg EP. 2019. Quorum Sensing Signal Selectivity and the Potential for Interspecies Cross Talk. mBio 10:10.1128/mbio.00146-19.
